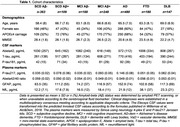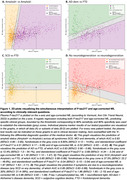# Use of a multimarker blood test in the heterogenous memory clinic setting: an updated and simplified interpretation tool

**DOI:** 10.1002/alz70856_106371

**Published:** 2026-01-09

**Authors:** Inge M.W. Verberk, Michelle C. Barboure, Sinthujah Vigneswaran, Lynn Boonkamp, Calvin Trieu, Elsmarieke van de Giessen, Afina W. Lemstra, Yolande A.L. Pijnenburg, Wiesje M. van der Flier, Martijn Schut, Anouk den Braber, David H Wilson, Argonde C. van Harten, Charlotte E. Teunissen

**Affiliations:** ^1^ Neurochemistry Laboratory, Department of Laboratory Medicine, Amsterdam UMC, Vrije Universiteit Amsterdam, Amsterdam Neuroscience, Amsterdam, Netherlands; ^2^ Alzheimer Center, Department of Neurology, Amsterdam UMC, Vrije Universiteit Amsterdam, Amsterdam Neuroscience, Amsterdam, Netherlands; ^3^ Department of Radiology and Nuclear Medicine, Amsterdam UMC, Vrije Universiteit Amsterdam, Amsterdam Neuroscience, Amsterdam, Netherlands; ^4^ Alzheimer Center Amsterdam, Department of Neurology, Amsterdam UMC, location VUmc, Amsterdam, Netherlands; ^5^ Amsterdam Public Health, Methodology & Digital Health, Amsterdam UMC, Vrije Universiteit Amsterdam, Amsterdam, Netherlands; ^6^ Translational Artificial Intelligence laboratory, Department of Laboratory Medicine, Amsterdam UMC, Vrije Universiteit Amsterdam, Amsterdam, Netherlands; ^7^ Biological Psychiatry, Vrije Universiteit Amsterdam, Amsterdam, Netherlands; ^8^ Quanterix Corp., Billerica, MA, USA

## Abstract

**Background:**

Patients referred to specialized memory clinics present with different symptoms and etiologies. A blood test could streamline establishing etiological diagnoses. Given the diversity in patients seen at memory clinics, relying on a single marker, e.g. phosphorylated tau217 (pTau217), is insufficient. However, multi‐marker interpretation in the clinical context is challenging. We aim to provide a comprehensive multi‐marker interpretation tool.

**Method:**

We follow up on our published work (Verberk, Alz&Dem, 2024) in which we presented a result visualization approach for *p*‐tau181, glial fibrillary acidic protein (GFAP) and neurofilament light (NfL). We now extended our cohort to *n* = 1773 Amsterdam Dementia Cohort participants with diagnosis established by clinical consensus according to applicable criteria (table 1) and measured *p*‐tau217 (Simoa Janssen) in addition to amyloid‐beta (Abeta)_42/40_, GFAP and NfL (age‐corrected). Starting point of the data analyses were clinically relevant questions: 1) identify Amyloid+ across all syndromes SCD, MCI and dementia, 2) discriminate FTD from AD, 3) discriminate FTD from any SCD (Amyloid+/‐), 4) predict if symptoms are due to a neurodegenerative disease (any dementia vs any SCD). We performed logistic regression analyses with 100‐fold Monte‐Carlo cross‐validation across 70%‐30% train‐test splits for all plasma marker combinations, and selected the simplest and most effective combination, based on number of markers, AUC, marker importance and %individuals falling in the grey zone (bounded by 90%‐sensitivity and 90%‐specificity thresholds). We subsequently developed a visualization tool.

**Result:**

The simplest and most effective combination of plasma biomarkers across the clinical questions was *p*‐tau217 with age‐corrected NfL, with AUCs ranging from 0.85 (FTD/SCD) to 0.93 (Amyloid+/Amyloid‐). %individuals in the grey zones ranged from 8.9% (Amyloid+/Amyloid‐) to 37.5% (FTD/SCD). Figure 1 shows our visualization tool: plasma values of our tested population, the size of the grey zones, and the influence of both plasma markers on the outcome prediction (angle of the grey zone) can be appreciated, per question. Plasma results of new patients are plotted on these graphs, to inform on their etiological diagnosis.

**Conclusion:**

Plasma marker results of new patients are easily interpretable with our visualization tool. Grey zones are generally small, benefitting the confidence of plasma biomarker use in routine dementia practise.